# Antiviral Potential of Azathioprine and Its Derivative 6- Mercaptopurine: A Narrative Literature Review

**DOI:** 10.3390/ph17020174

**Published:** 2024-01-30

**Authors:** Carolina Rios-Usuga, Marlen Martinez-Gutierrez, Julian Ruiz-Saenz

**Affiliations:** 1 Grupo de Investigación en Ciencias Animales—GRICA, Facultad de Medicina Veterinaria y Zootecnia, Universidad Cooperativa de Colombia, Bucaramanga 680002, Colombia; carolina.rios@udea.edu.co (C.R.-U.); marlen.martinez@udea.edu.co (M.M.-G.); 2 Grupo de Investigación en Microbiología Veterinaria, Escuela de Microbiología, Universidad de Antioquia UdeA, Medellín 050001, Colombia

**Keywords:** azathioprine, antimetabolite, autoimmune, hepatotoxicity, antiviral

## Abstract

The use of azathioprine (AZA) in human medicine dates back to research conducted in 1975 that led to the development of several drugs, including 6-mercaptopurine. In 1958, it was shown that 6-mercaptopurine decreased the production of antibodies against earlier administered antigens, raising the hypothesis of an immunomodulatory effect. AZA is a prodrug that belongs to the thiopurine group of drugs that behave as purine analogs. After absorption, it is converted into 6-mercaptopurine. Subsequently, it can be degraded through various enzymatic pathways into inactive compounds and biologically active compounds related to the mechanism of action, which has been the subject of study to evaluate a possible antiviral effect. This study aims to examine the metabolism, mechanism of action, and antiviral potential of AZA and its derivatives, exploring AZA impact on antiviral targets and adverse effects through a narrative literature review. Ultimately, the review will provide insights into the antiviral mechanism, present evidence of its in vitro effectiveness against various DNA and RNA viruses, and suggest in vivo studies to further demonstrate its antiviral effects.

## 1. Introduction: Brief History and Development of Azathioprine 

The use of azathioprine (AZA) in human medicine dates back to research conducted in 1954 by Nobel Laureates George Herbert Hitchings and Gertrude Elion, who studied the nucleic acid metabolism in normal cells, tumoral cells, and bacterial cells, with the goal of interfering with the purine pathway to block the production of nucleotides such as adenine and guanine, thereby inhibiting DNA synthesis and cell replication [1,2]. Those early works, led to the development of drugs that are still in use in clinical practice such as allopurinol, acyclovir, trimethoprim, and 6-mercaptopurine (6-MP) [3]. This review details the metabolism of AZA and the mechanism of action of its derivatives as a possible antiviral therapeutic agent, and it discusses studies conducted to evaluate the antiviral effect of AZA and its derivatives on both, DNA and RNA viruses.

Mercaptopurines were primarily evaluated for the treatment of leukemia in the early 1950s. The original studies on this compound in mice showed tumor inhibition against Sarcoma 180 [4,5] and effectively inhibited the growth of adenocarcinoma EO771, Bashford carcinoma 63, carcinoma 1025, epidermoid carcinoma, among others [6]. Extensive toxicologic and pathologic studies of 6-MP were developed in mice, rats, cats, and dogs [5,7]. The first human trial developed by Burchenal et al., was published in 1953, involving the use of 6-MP in the treatment of multiple leukemias and other cancers, in children and adults, inducing good clinical and hematologic remissions and clinical improvement [8].

**Figure 1:**
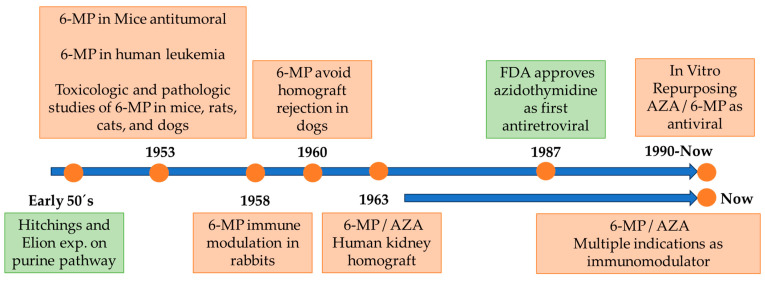
Timeline of 6-MP/AZA development. Green boxes denote key facts not directly related to the 6-MP/AZA. See text for details.

Later, Robert Schwartz in 1958 showed that treatment with 6-MP in rabbits led to a decrease in the generation of antibodies against previously administered antigens, which raised the hypothesis of an immunomodulatory effect and its use in transplants [9]. This research led to the first report of the positive effect of 6-MP on the rejection of dog kidney homograft, increasing patient survival [10], and soon to the human studies involving the use of 6-MP and azathioprine instead of irradiation in patients with kidney homografts [11] (Figure 1).

After the identification of human retroviruses such as the human immunodeficiency virus (HIV-1), research efforts were carried out to identify drugs capable of treating or preventing lethal diseases caused by viruses, and it was shown that several nucleoside analogs exhibit in vitro anti-HIV-1 activity in accordance with the development of clinical trials in 1987 [12]. It was also found that a variety of purine derivatives had antiviral potential, such as hypoxanthine (6-hydroxypurine), which has strong antitumor activity with minimal side effects. In addition, this compound is an intermediate product in the synthesis of other substituted purines, such as 6-mercaptopurine, considered an antiviral agent of the purine series and derived from AZA [13].

**Figure 2:**
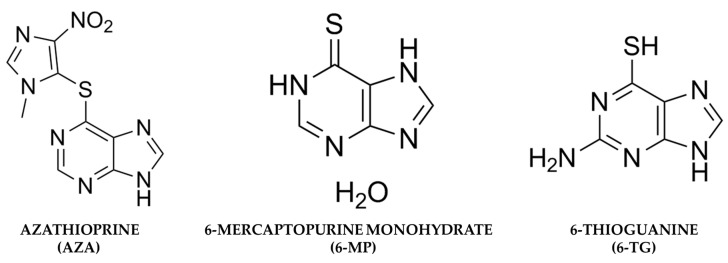
Chemical structures of azathioprine, 6-mercaptopurine, and 6-Thioguanine.

AZA is a prodrug that belongs to a group of immunosuppressive drugs called thiopurines, which behave like purine analogs. Although AZA and 6-MP (Figure 2) have been studied with the same therapeutic objective, it has also been shown that the bioavailability of AZA could be higher than that of 6-MP. When 6-MP is administered orally, it subsequently undergoes extensive catabolism by the enzyme xanthine oxidase (XO), which can be found in the liver and intestinal mucosa, and by thiopurine methyltransferase (TPMT), which is present in the intestinal mucosa and methylates 6-MP into inactive metabolites, limiting its systemic bioavailability [14]. After being taken orally, AZA exhibits an approximate 88% bioavailability and undergoes rapid absorption. Around 30% of it binds to plasma proteins, and it achieves its highest serum concentration approximately two hours post-ingestion [15].

## 2. Azathioprine Metabolism

After its absorption through the in the intestinal wall of the gastrointestinal tract, liver, and red blood cells, AZA is metabolized into 6-mercaptopurine (6-MP) due to nonenzymatic removal of its nitroimidazole ring, which seems to be mediated by glutathione S-transferase [2,8,9,10]. Subsequently, 6-MP is converted by different enzyme systems: xanthine oxidase (XO), thiopurine S-methyltransferase (TPMT), and hypoxanthine-guanine phosphoribosyltransferase (HGPRT). The XO catalyzes the conversion of 6-MP into a pharmacologically inactive metabolite called 6-thioric acid (6-TUA), while the enzyme TPMT forms 6-methylmercaptopurine (6-MMP) from 6-MP, and the enzyme HGPRT is responsible for transforming 6-MP into 6-thioinosine 5′-monophosphate (6-TIMP). 6-TIMP is catabolized by the enzymatic action of inosine monophosphate dehydrogenase (IMPD) into 6-thioxanthosine 5′-monophosphate (6-TXMP), which in turn can be transformed into 6-thioguanosine 5′-monophosphate (6-TGMP) via the enzyme guanosine monophosphate (GMPS). 6-TGMP can be converted to 6-thioguanosine 5′-diphosphate (6-TGDP) and 6-thioguanosine 5′-triphosphate (6-TGTP) by two respective kinases. Within the nucleotide pool of 6-thioguanine (6-TGN), 6-TGTP (approximately 80% of TGN) and 6-TGDP (approximately 16% of TGN) are the main metabolites, while 6-TGMP is present only in traces. Therefore, 6-TGN levels are strongly correlated with 6-TGTP and 6-TGDP concentrations. 6-TGN has an average half-life of 5 days (range between 3–13 days), and all of them can be methylated by TPMT [3,16,17,18]. Recently, it has been demonstrated that an unusual AZA hydrolysis occurs in the presence of ruthenium, thus generating hypoxanthine instead of the expected 6-MP antimetabolite [19]. Hypoxanthine (6-hydroxypurine) is a deaminated form of adenine, and a constituent of the nucleoside inosine is formed during purine metabolism. It is found in both tissues and body fluids of human beings and animals, and it has been proposed as a biomarker for a variety of disease such as hypoxia, colorectal cancer, Alzheimer’s disease, multiple sclerosis, and cardiac ischemia [19].

Additionally, 6-TIMP is methylated by TPMT to subsequently form 6-methylthioinosine 5′-monophosphate (6-MTIMP), 6-methylthioinosine 5′-diphosphate (6-MTIDP), and 6-methylthioinosine 5′-triphosphate (6-MTITP). These last three metabolites are called 6-methylmercaptopurine ribonucleotides (6-MMPR). 6-TIMP can also be phosphorylated by the enzyme monophosphate kinase to form 6-thioinosine 5′-diphosphate (6-TIDP). Then, the enzyme diphosphate kinase forms 6-thioinosine 5′-triphosphate (6-TITP) and finally returns to 6-TIMP after an enzymatic reaction catalyzed by the enzyme inosine triphosphate pyrophosphatase (ITPase) [17,20,21].

Regarding the metabolism of 6-TG, which is less complex than that of 6-MP and AZA, the absorption by the oral administration of 6-TG is incomplete and highly variable, generating a bioavailability of 14 to 46%, unlike absorption in the form of a prodrug (AZA) that can increase approximately 80% [17,20,21], as has been reported previously [15]. Both 6-MP and 6-TG are taken up by the HGPRT enzyme, which participates in the synthesis of nucleotide salvage from immune effector cells and is converted into their respective nucleoside monophosphate (TIMP and TGMP). In addition to the competitive catabolic reactions, TPMT by means of S-methylation inactivates 6-MP and 6-TG, and it XO converts 6-MP into 6-thiouric acid, which is an inactive compound.

Methylated TIMP (meTIMP), but not meTGMP, is an effective inhibitor of de novo purine biosynthesis. TIMP, which escapes catabolism, is further metabolized by inosine monophosphate dehydrogenase (IMPDH) and guanine monophosphate (GMPS) to TGMP. The sequential action of deoxynucleoside kinases and reductase generates thio-GTP and thio-dGTP, which are the substrates for the incorporation of 6-TG into RNA and DNA [17,20]. The active metabolite of 6-TG inserts as an analogous base in DNA and RNA nucleic acids, inhibiting DNA repair, replication, and transcription [16,21].

Thiopurines enter the cells, by the membrane transporters for the respective nucleosides, which are then phosphorylated to the forms of nucleotides by the respective kinases mentioned above. Once the different forms of nucleotides are obtained, the step initial phosphorylation (flow regulator of the process) is through a nucleoside kinase, which leads to the production of a nucleotide monophosphate metabolite. Subsequently, a second phosphorylation step is carried out by the nucleotide monophosphate kinase, which generates a diphosphate nucleotide, and the third phosphorylation step is carried out by the nucleoside diphosphate kinase, which generates a nucleotide triphosphate. These forms can be incorporated into nucleic acids in competition with their homologous components, or they can inhibit nucleic acid synthesis by inhibiting essential enzymes such as polymerases [22,23].

When administering drugs, such as AZA, for a long time, it is important to bear in mind that they can generate toxicity, which is associated with two possible mechanisms: the first is the alteration in the cellular repair systems associated with DNA, specifically the alteration mediated by PCNa (proliferating cell nuclear antigen), for DNA polymerase-δ and the repair factor MutLα, MutL- exonuclease. The presence of a miscoded base analog (in this case, methyl 6-thioguanine (me6-TG) and methyl O6-guanine (O6-meG)) in the template chain can alter the repair system. Here, me6- Synthesized TG or O6-meG produced by a methylating agent directs the incorporation of thymine (T), forming Me6-TG:T or O6-meG:T. This condition activates MutSα-MutLα (cell repair factor), which triggers correction attempts that remain incomplete due to the impossibility of incorporating a base that can adequately complement the erroneous base. Consequently, structural alterations of DNA generated by incomplete repair attempts can be observed as breaks in DNA strands during the S phase of the next cell cycle, which become potentially lethal aberrant DNA structures that are substrates for recombination. This triggers the activation of the ATR proteins (ataxia telangiectasia and relation with Rad3) and ATRIP (protein that interacts with ATR), which are responsive to DNA damage with consequent signaling for phosphorylation of CHEK1 (serine/threonine kinase), which regulates phosphorylation of Cdc25 (threonine and tyrosine phosphatase) and thus the arrest of the G2 cell cycle [23]. The second mechanism is related to the ability to absorb ultraviolet radiation (UV), since thiopurines are nucleotides that have a superior capacity in the capture of ultraviolet radiation, which generates a considerable increase in the generation of reactive oxygen species (ROS) [17,23].

The various radical and nonradical types of ROS have the potential to induce direct harm to the components of DNA by creating oxidized bases and deoxyribose. Additionally, these ROS variants can cleave the phosphodiester backbone, leading to the formation of breaks in the DNA strand, either as single-stranded or double-stranded breaks. In addition, they can initiate reactions that generate secondary reactive species, such as aldehydes, which can also form DNA lesions. 6-Thioguanine is a very important target for oxidation, generating one of the main products in this process (G^SO3^, guanine 6-sulfonate). Proteins are also vulnerable to ROS, particularly those ^1^O_2_ that react with aromatic, basic and sulfur amino acids, altering the three-dimensional patterns of peptides and proteins, generating alterations in their function and secondary cellular damage [16,17,23].

ROS originate from two distinct types of reactions. In Type I reactions, thiopurine absorbs UVA energy, transitioning to an unstable excited triplet state. This state can then react with oxygen, resulting in the formation of a radical of thiopurine and superoxide (·O_2_). Subsequently, in a Fenton-type reaction, (·O_2_) is transformed into hydroxyl radicals (·OH), which pose a threat to cellular macromolecules. In Type II reactions, energy is directly transferred from absorbed radiation to ground-state triplet molecular oxygen (^3^O_2_), generating singlet oxygen (^1^O_2_), a nonradical with a longer half-life compared to most ROS. Both ·OH and ^1^O_2_ have the potential to cause mutagenic damage to DNA, encompassing strand breakage and oxidation-induced alterations in the constituent nitrogenous bases and deoxyribose [22,23].

**Figure 3:**
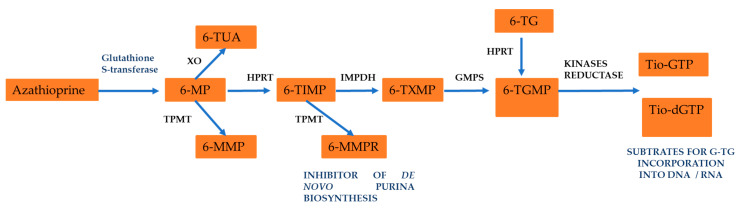
Azathioprine metabolism in orally administration. See text for details.

In Figure 3, this metabolic process is presented, where the active compounds mentioned above have been related to the immunosuppressive effect and possible hepatotoxic effects in the patient treated with AZA.

The immunosuppressive mechanism of action of thiopurines has not been fully clarified in 40 years of use, but the recognition of its metabolites has allowed us to determine its possible mechanism. MeTIMP is an inhibitor of the de novo synthesis of purines because it inhibits the synthesis of ADT and GTP, exerts an immunosuppressive effect, and blocks the proliferation of lymphoid cells. By the action of HGPRT, 6-TGN is converted into 6-thioguanosine 5-monophosphate (TGMP), which by the action of kinases and reductases forms deoxy-6-thioguanosine 5-triphosphate (dGS), that is incorporated into DNA and is able to stop the cell cycle and promote apoptosis [15].

Apoptosis is the final mechanism by which thiopurines exert their immunosuppressive effect [24]. There are two main signaling pathways (extrinsic and intrinsic) that control the initiation of apoptosis in mammals, which involve the participation of specialized proteolytic enzymes and caspases. Members of these protein families can be divided into antiapoptotic (Bcl-2 and Bcl-xL) and proapoptotic (Bax and Bak) proteins. Extrinsic pathways involve the participation of cell surface receptors belonging to the tumor necrosis factor (TNF) superfamily and the consequent activation of caspase 8. The intrinsic cellular pathway involves the alteration of the mitochondrial membrane by pro-apoptotic members of the Bcl-2 family and the consequent release of factors, such as cytochrome c, that promote the activation of caspase-9. The activation of these caspases leads to the cleavage of a set of proteins, resulting in a characteristic pattern of gene expression and cell disassembly. Selective membrane permeability (MMP) is an important mechanism underlying cell death, where mitochondrial disruption culminates in a rapid loss of this mitochondrial membrane potential, a release of cytochrome c, and cell death [15].

In functional studies, it has been shown that AZA uses a caspase 9-dependent mitochondrial pathway that involves the negative relationship of Bcl-xL and the suppression of the mitochondrial membrane potential; the blockade of caspase 9 inhibits the apoptosis induced by AZA. These studies have shown that AZA and its derivatives 6-MP and 6-TG induce apoptosis in CD4 T lymphocytes in humans only in cells with activation of the Rac1 gene that is co-stimulated through CD28, which makes their use highly effective in the treatment of autoimmune diseases [24]. By inhibiting purine synthesis, AZA and 6-MP disrupts the proliferation of lymphocytes, which are key immune cells involved in the inflammatory process. This immunosuppressive effect helps modulating the aberrant immune response seen in autoimmune diseases like rheumatoid arthritis and inflammatory bowel diseases, and it is currently still being used in the treatment of systemic autoimmune disease, including overlap syndromes [3,25]. Also, by the same mechanism, interfering with the activation of T lymphocytes and blocking DNA synthesis in rapidly dividing immune cells AZA and its derivates in combination with other immunosuppressive drugs prevent allograft rejection after organ transplantation, by creating an immunosuppressive environment that allows the transplanted organ to integrate and function without being attacked by the recipient’s immune system [26,27].

In terms of the hepatotoxic and myelosuppressive side effects, it has been described as an idiosyncrasy linked to genetic variations of TPMT in humans, since TPMT activity is controlled by a common genetic polymorphism and one in 300 subjects has a very low enzymatic activity; this condition was first reported in 1989 in a study with adults who were administered AZA [28]. Very high serum levels of this enzyme cause the shunting of 6-MP to 6MMP, preferring its conversion to 6-TGN. Therefore, the serum concentration of 6-TGN nucleotides is reduced, thereby decreasing the myelosuppressive effect of AZA, and by increasing the serum concentration of 6MMP, the risk of hepatotoxicity increases [18]. The effects of AZA-induced myelosuppression related to TPMT deficiency have been documented in different groups of patients [29]. Although patients with deficiencies in TPMT synthesis should be treated with lower doses of thiopurines to avoid toxic concentrations of 6-TGN, these patients do not form 6-MeTIMP and can tolerate considerably higher concentrations of 6-TGN than patients with high levels of TPMT activity [15]. Likewise, it has established that polymorphisms of glutathione-S-transferase-M1 may influence AZA effects in young patients with inflammatory bowel disease by increasing the drug activation and by modulating oxidative stress and apoptosis [30].

However, TPMT deficiency does not absolutely contraindicate treatment with AZA or mercaptopurine. And, in humans, there are studies where TPMT measurements have been made to determine the doses of AZA to use and thus avoid a marked myelosuppression and/or hepatotoxic effect due to genetic variability in the amount of TPMT between individuals [18]. Similarly, in treatments that require the chronic use of AZA, it is necessary to perform a follow-up by measuring liver enzymes and serial hemograms to evaluate and avoid medical complications in the patient due to its side effects. And, although it has been reported that during AZA treatment there is an increase in serum aminotransferase (liver enzyme) levels, generally associated with high levels of 6-MP, the risk of severe liver dysfunction is low, and aminotransferase levels generally normalize a few weeks after discontinuation of treatment [31]. In canine studies, an increase in liver enzymes greater than twice the maximum values determined as normal parameters has been estimated in 15% of patients treated with AZA in the first two weeks, and thrombocytopenia or neutropenia in 8% of patients, which were evidenced approximately 6 weeks after starting treatment at an average dose of 1.9 mg/kg [32].

In veterinary medicine, regarding genetic variation associated with the amount of TPMT in dogs, it has been found that there are no associated differences between sex, age, and sterilization, but it was identified that giant Schnauzers have lower TPMT activity than other breeds and that Alaskan Malamutes have much higher activity, which could be an important factor to consider when evaluating the tolerability and efficacy of thiopurine treatment [33]. There are also reports where German Shepherds have shown a high risk of hepatotoxicity in the treatment with AZA [32].

Another important aspect that should be considered when performing therapy with AZA is the possible presentation of opportunistic infections, such as the presentation of disseminated cutaneous herpes zoster in humans, which has been described as a consequence of the infectious reactivation of the varicella zoster virus (VZV) that is latent in dorsal nodes in patients with rheumatoid arthritis with pharmacological immunosuppression [34] or immunocompromised by other infectious agents, such as HIV-1 [35]. However, in a 2010 study that sought to determine herpes zoster infection from 1145 patients with systemic lupus erythematosus (SLE), zoster was diagnosed in 51 patients with SLE (4.45%), where only 39.2% were administered AZA, the main trigger of viral infection being the concomitant treatment of corticosteroids and immunosuppressants, and not the active disease [36]. Recently, it has been reported that AZA therapy selectively induced pronounced NK cell depletion and concomitant IFN-γ deficiency plus a specific contraction of classical TH1 cells leading to an increased occurrence of reactivation of endogenous latent herpesviruses [37].

AZA is commonly used in dogs for the treatment of some immune-mediated diseases, such as autoimmune hemolytic anemia, immune-mediated thrombocytopenia, rheumatoid arthritis, immune-mediated polyarthritis, inflammatory bowel disease, Evans syndrome, dermatological diseases, and other immune-mediated diseases [32,33]. In cats, AZA it is not used often due to the frequent appearance of bone marrow suppression evidenced in patients treated at an average dose of 2 mg/kg for intermediate days. This treatment often results in a hypocellular marrow with a significant reduction in the myeloid series, and even leads to respiratory tract infections [38].

## 3. AZA and Its Derivatives as Antiviral Agents

Considering the previously mentioned mechanisms of action of azathioprine and its derivatives, studies have been carried out to assess its antiviral effect in vitro and in vivo with different viruses (Table 1). Antimetabolite immunosuppressants have some similarities with previously identified antivirals such as ribavirin (RIB), which inhibits inosine monophosphate dehydrogenase (IMPDH) [39] as does mycophenolate acid (MPA). In addition, it has been shown that in AZA metabolism, meTIMP concentrations are inversely correlated with IMPDH activity [40].

### 3.1. Herpesviruses

In 1991, Shiraki et al. designed a study to characterize the effects against HCMV (human cytomegalovirus), HSV (herpes simplex virus), and VZV (varicella zoster virus) of AZA in vitro and any beneficial effects of AZA in combination therapy with other immunosuppressants in transplant recipients, since the same authors had previously shown that cyclosporine (CsA) and prednisolone (Pred) improved the replication of HCMV in vitro, while AZA suppressed it at immunosuppressive concentrations [42]. In another study by the same authors, it was found that AZA at a dose of 0.592 µg/mL exhibited antiviral activity against HCMV in vitro with a 50% reduction in plaques in human embryonic lung (HEL) cells, as well as HSV and VZV at doses >20 µg/mL, where the AZA dose for the 50% inhibition of plaque growth in HEL was 30 µg/mL.

Hence, the researchers concluded that AZA exhibits potent anti-HCMV activity at the concentrations utilized for immunosuppression. Moreover, when cells infected with HCMV were concurrently treated with CsA at 0.2 µg/mL and Prednisone (Pred) at 0.3 µg/mL along with AZA, the doses of AZA required for a 50% reduction in plaque formation were lowered to 0.73 µg/mL. This suggests that the combination of AZA with CsA and Pred not only achieves effective immunosuppression but also effectively suppresses HCMV infection in transplant recipients [41].

### 3.2. Flaviviruses

In 2004, researchers wanted to evaluate the antiviral effect of both MPA and AZA in the treatment of hepatitis C virus (HCV), but due to its high genotypic variability and other variables associated with the virus, both the understanding of its replicative cycle and the antiviral therapy study have been hampered by the lack of a satisfactory cell culture system [52], for which they conducted a study with other flaviviruses genomically related to HCV and belonging to the same viral family, such as bovine viral diarrhea virus (BVDV), which was cultured in bovine kidney cells (MDBK) using BVDV type 1. When measuring the growth of BVDV in cells exposed to both compounds, both MPA and, to a lesser extent, AZA showed cytostatic and toxic effects, being much higher for MPA since even at very low concentrations, all the cells died. The antiviral effect of AZA was greater than that of MPA, with only 12% of the virus produced in cells grown in the presence of AZA compared to the amount of virus produced per cell grown in the presence of MPA. Additionally, the decrease in cell growth caused by AZA at very high concentrations can be prevented with high concentrations of thymidine (1Mm), because DNA synthesis is limited and the toxic metabolite of AZA is not produced or incorporated (6-TGN) in cellular DNA, thus reducing cytotoxicity but preserving the decrease in viral replication [43].

In 2004, a prospective crossover study with a 9-month follow-up was conducted to compare the impact of mycophenolate mofetil (MMF) and AZA on allograft function and viral load in 13 liver transplant recipients with chronic recurrent HCV infection. At the study beginning, the patients exhibited a mean viral load of 0.74 × 10^6^ ± 0.47 × 10^6^ copies of mRNA/mL. Among those who continued MMF treatment, the mean viral load at the end of the MMF period increased to 1.64 × 10^6^ ± 1.34 × 10^6^ copies of mRNA/mL compared to baseline. Notably, 11 out of 13 patients experienced an increase in viral load during the MMF period, while the remaining 2 patients showed slight decreases. Upon reintroducing AZA therapy in the last 3 months of the study, the mean viral load started to decline, reaching 1.58 × 10^6^ (±2.85 × 10^6^) copies of mRNA/mL at the end of the following, although this decrease was not statistically significant. Liver enzyme alterations were generally not associated with viral load increases, except for two patients who exhibited variations in liver function tests, one due to biliary stenosis and the other associated with severe facial infection by VZV. Despite the study’s limited sample size, the authors were unable to demonstrate a long-term antiviral effect of MMF in immunosuppressed patients with chronic recurrent hepatitis C. Nevertheless, they did show that substituting azathioprine for mycophenolate offered no advantage in long-term HCV-infected recipients, and stability was maintained with azathioprine-based maintenance immunosuppression [53].

In 2008, another study was carried out where it was evaluated in cultures of MDBK cells inoculated with BVDV, where the following metabolites were evaluated: 6-mercaptopurine (6-MP), 6-thioinosine (6-TI), 6-thioguanosine riboside (6-TGr), 6-methylmercaptopurine riboside (6MMPr), sulfasalazine, adenine, adenosine, inosine, and guanosine. To determine which AZA metabolite has the most potent antiviral activity and to better understand how this compound can act in vitro, the authors were able to show that 6-TI and 6-MP show a similar antiviral activity, although 6-TI is slightly more potent than 6MP at 5 µM. 6MMP administered as a base had little activity, although with the addition of a riboside to produce 6MMPr, the inhibition of BVDV increased more than 500 times, showing 6MMPr as the most potent antiviral with an IC 90 at a concentration of 1.0 μM. Decreasing almost 100 times BVDV viral titers, while 6-TGr did not reach IC 90 even at such high concentrations. At 50 µM, 6-TI had an IC 90 value of 2.9 µM. Furthermore, all thiopurines showed similar inhibition of cell growth, and no statistically significant variations in cytostatic effects were observed among any of the treated cells, although 6-TGr exhibited cytostatic effects similar to those of 6MMPr and had little or no antiviral activity at 10 µM or even at very high doses. All thiopurines showed a cytotoxic concentration 50 (CC50) greater than 500 µM [44].

Additionally, to test whether 6-TI could be converted to 6MMPr to produce an antiviral effect, TPMT activity was inhibited in cells using sulfasalazine, and the concentrations of 6-TI and 6MMPr were kept constant at 1 μM in a range of concentrations of sulfasalazine, where they found that sulfasalazine decreased the antiviral effect of 6-TI depending on the dose but showed little effect on the activity of 6MMPr. To measure the effects of purine mixtures on viral replication, guanosine, inosine, or adenosine was added during BVDV infection. By adding guanosine, the viral titers were reduced in the presence of 6MMPr below the detection limit of the plaque assays used, the increase in inosine concentrations increased the inhibition of BVDV by 80 times both by 6-TI and by 6MMPr, the inhibition of 6MMPr was reduced almost 10 times and 6-TI 25 times in the presence of inosine, and adenosine reduced the inhibition of 6MMPr five times and reduced the inhibition of 6-TI 40 times. Similarly, researchers using Ntat2ANeo cells containing an HCV replicon evaluated treatment with increasing concentrations of 6-TI and 6MMPr to measure the effects of thiopurines on HCV replication and found that 6-TI had little anti-HCV activity, while 6MMPr inhibited the production of SEAP (secreted alkaline phosphatase) almost 10 times [44].

After the previous study, the same group of researchers carried out another study where they used cultures of VERO and BHK-21 cells, HEK293T, HepG2, Huh7, Huh6, and cells of Aedes albopictus (C6/36), inoculated with different flaviviruses: western virus Nile virus (WNV) of a New York strain, Dengue virus type 2 (DENV-2) strain New Guinea, strain DENV-2 16681, and strain PFLYF17D of Yellow Fever virus (YFV), using 6MMPr as an antiviral compound where it was evidenced that 6MMPr inhibited the viral production of DENV-2 and YFV approximately 10 times in Huh7 and Huh6 culture, 100 times in HepG2, and 10,000 times in HEK293T; this inhibition in HEK293T was not correlated with cytotoxicity, which was consistent with the results of the previous investigation in 2008, where 6MMPr up to 500 μM did not cause cytotoxicity. At all concentrations tested, 6MMPr inhibited DENV-2 viral replication approximately 10 times more than WNV at 48 h and 72 h PI. In addition, it was shown that 6MMPr caused 10- to 100-fold inhibition in HEK293T and VERO cells compared to Huh7 and Huh6 cells. Finally, they explored the use of 6MMPr as an antiviral therapy for WNV in an immunocompetent C3H mouse model using a dose of 0.25 mg or 0.5 mg of 6MMPr (25 mg or 50 mg/kg/day) once a day and twice a day. During 7 and 10 consecutive days, where a weight loss of more than 10% was observed in all the mice in the high-dose group and half in the low-dose group, all the mice inoculated with WNV treated with vehicle and 6MMPr showed signs of disease, and there were no significant differences in morbidity and mortality between all the individuals treated and not treated with 6MMPr. In addition, mild toxicity manifested as weight loss. When obtaining these results, they included a group pretreated with 0.5 mg of 6MMPr once a day for 7 days, starting one day before inoculation, and the treated group obtained a viral titer two times lower at the peak of viremia than the group without treatment but with a nonsignificant difference. They also evidenced higher viral loads in the brain than in the spinal cord, the site of inoculation, the lymph nodes and the spleen, indicating that the effect of 6MMPr is tissue-dependent in the in vivo mouse model [46].

In 2017, the antiviral activity and cytotoxicity of 6MMPr were evaluated against the epidemic strain of the Zika virus (ZIKV) that circulates in Brazil, which has been associated with neurological manifestations and congenital defects [54]. The study was carried out using VERO and SH-SY5Y cell cultures (human neurons). In this test, a reduction in the production of ZIKV greater than 99% was obtained in both cell lines, observing a CC50 at 291 µM in Vero cells, 460 µM in SH-SY5Y cells, an inhibitory concentration of 50 (IC50) at 24.5 µM and 20.3 µM, respectively, and a selectivity index (IS) for VERO cells of 11.9 and 22.7 for GH-SY5Y cells. This index highlights the safety profile of the drug for neuronal cells. These results identify, for the first time, the thiopurine nucleoside analog 6MMPr as a promising antiviral candidate for ZIKV in an in vivo assay [48].

### 3.3. Morbilliviruses

In 2017, the antiviral effect of 6MMPr and ribavirin (RIB) on canine distemper virus (CDV) was compared using a field strain in VEROdogSLAM cell culture. When we evaluated the cytotoxicity of both compounds, it was determined that 6MMPr was 3.3 times less toxic than RIB, where the CC50 value of 6MMPr was 1409 µM and that of RIB was 424 µM. In the analysis of antiviral activity by assessing the number of copies of CDV RNA, 6MMPr reduced the number of copies at 94% at high concentrations of 169 and 338 µM, while RIB showed lower antiviral effects with virus inhibition by 60% at the highest concentration of 81.5 μM [49].

### 3.4. Pneumoviruses

In 2019, a group of researchers in Beijing evaluated several active compounds against human respiratory syncytial virus (RSV) using a recombinant virus that expresses mGFP (RSV-mGFP) through the application of a high-throughput screening test (HTS). The HTS assay was validated by establishing two known RSV replication inhibitors (P13 and ribavirin), where when analyzing 2000 bioactive compounds and 52,800 synthesized compounds, they identified 62 compounds that presented an inhibition ≥50% for RSV, of which AZA and 6-MP were chosen together with ribavirin and P13 to be subsequently tested in vitro in culture of HEp-2 cells inoculated with recombinant RSV-mGFP and wtRSV (wild-type strain), demonstrating an IC50 of 6.69 ± 1.41 and 3.13 ± 0.98 µM for AZA and 6-MP, respectively, and a CC50 of 297.77 ± 23.93 and 183.73 ± 30.17 µM, respectively, with both compounds exhibiting a selectivity index (SI) exceeding 50. Additionally, AZA and 6-MP exerted inhibitory effects on the transcription and/or replication of the respiratory syncytial virus (RSV) genome. This observation aligns with the findings of the subsequent RSV minigenome assay, where BHK/T7 cells were cotransfected with the RSV minigenome plasmid pBR322B-RSV-Gluc and four helper plasmids. After 24 h, Gluc expression in the cell supernatant was detected. In comparison to the negative control group, both AZA and 6-MP demonstrated the ability to hinder Gluc expression, indicating their involvement in the replication/transcription phase of the RSV genome. [50].

The likely antiviral effect of AZA and its derivatives, including MeTIMP, is associated with their mechanism of action. MeTIMP works by inhibiting the synthesis of ADT and GTP, leading to the suppression of de novo purine synthesis [43,46,49]. The effect mediated by the derivative 6-TGN, which then by action of some kinases and reductases forms dGS, is capable of being incorporated into DNA to subsequently alter cell replication [15]. It is well known that this is the same machinery used in viral replication within the host cell.

### 3.5. Coronaviruses

In in vitro studies on various coronaviruses, it has been identified that 6-MP and 6-TG competitively, selectively, and reversibly inhibit SARS-CoV PLpro [45], This SARS-CoV PLpro is part of a group of cellular desubiquitinating enzymes (DUBs), essential for the viral replication cycle, which makes it a therapeutic target. In addition, it was evidenced that the inhibition is selective because they did not exert significant inhibitory effects against other proteases [47]. Similarly, it was evaluated whether mycophenolic acid, 6-MP, and 6-TG can synergistically inhibit MERS-CoV PLpro, where it was found that a noncompetitive inhibitor (mycophenolic acid) and two competitive inhibitors (6-MP and 6-TG) independently and synergistically inhibit the proteolytic activity and desubiquitination of MERS-CoV PLpro in *E. coli* BL21 cells [35]. In another study to investigate whether 6-MP and 6-TG could be potential inhibitors of other cellular or viral DUBs, a new search using SSM to find structures similar to PLpro in the Protein Data Bank (PDB), indicated that the interactions of thiopurines with different DUBs vary significantly, suggesting that it is possible to develop selective inhibitors targeting individual DUBs [55].

### 3.6. Influenzaviruses

On the other hand, the influenza A virus (IAV) uses host mechanisms to limit the expression of antiviral genes and redirect the machinery to the synthesis of viral proteins. The replication of IAV is sensitive to inhibitors of protein synthesis that block the initiation of translation and induce the formation of stress granules (SGs), which are cytoplasmic condensates of untranslated messenger ribonucleoprotein complexes [56]. In an investigation utilizing an automated antiviral drug assessment system, researchers discovered that 6-thioguanine (6-TG) and 6-thioguanosine (6-TGo) induced the formation of stress granules (SGs) in IAV-infected cells, impeding their replication. Both 6-TG and 6-TGo specifically disrupted the synthesis and maturation of IAV glycoproteins, namely hemagglutinin (HA) and neuraminidase (NA), while leaving the levels of viral RNAs encoding these proteins unaffected. Importantly, these thiopurines had minimal impact on other IAV proteins or the overall synthesis of host proteins [51]. These studies show an antiviral effect of AZA derivatives, associated with the mechanism of action of selective inhibition of viral protein synthesis.

## 4. Concluding Remarks and Future Prospects

**Figure 4:**
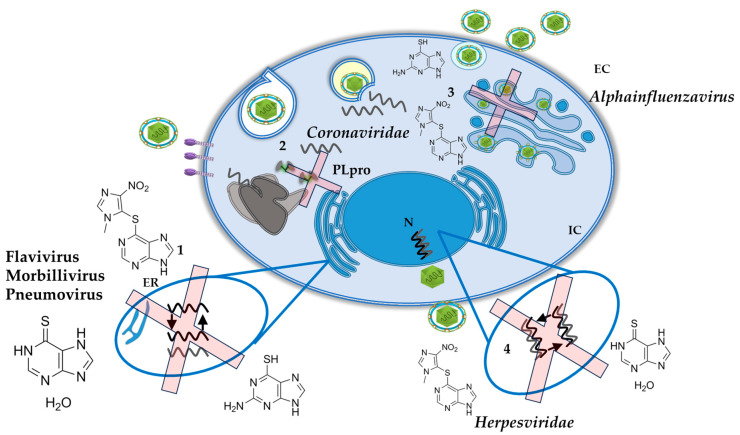
Schematic view of the potential antiviral targets of AZA and its derivates on different viruses. (1). Inhibition of replication in RNA Viruses. (2). Inhibition of PLpro for Coronaviruses. (3). Activation of the unfolded protein response sensors for Influenza A virus. (4). Inhibition of replication in human herpesviruses. See text for details.

Given the scientific evidence of the metabolism of AZA and its derivatives, plus the studies that report a potential inhibitor of viral replication, mainly in viruses with RNA genome, AZA and its derivates could be included in the long list of potential medications for drug repurposing. Based on the revised literature, we could speculate that AZA and its derivates exert their antiviral effects, in most of the viruses, via a decrease purine synthesis pathway leading to a depletion of the intracellular nucleotide pool and decreasing the availability of nucleotides for the synthesis of RNA and DNA viruses (Figure 4). This also includes other pathways such as the inhibition of PLpro for Coronaviruses, and the activation of the unfolded protein response sensors for Influenza A virus. These non-common pathways need to be further evaluated.

Nucleotide metabolism is one of the more exploited host processes for the development of antiviral targets for several human viruses via design or repurposing [57]. Several cellular enzymes of the pathways for nucleotide biosynthesis have been successfully tested with promising inhibitors for different viruses [58]. Nucleosides and nucleotides as Direct Acting Antivirals (ATC: J05A) includes various compounds that can inhibit enzymes involved in purine synthesis. These drugs disrupt the ability of the viruses to replicate its genetic material such as mycophenolate, mofetil, and ribavirin, among others.

However, AZA does not belong to this group; AZA’s main therapeutic objective is immunosuppressive (ATC: L04AX01—antineoplastic and immunomodulating agents) and its approval goes in this therapeutic use. The evidence as antiviral in vitro is extensive, and the doses used for antiviral effect are lower immunosuppressive doses, which could favor the antiviral effect without the side effects of immunosuppression or hepatotoxicity. For AZA to become an antiviral approved drug, it is necessary to clarify the mechanism of antiviral action and strengthen the evidence that promote in vivo studies and clinical trials, especially in viruses that do not have an established therapeutic protocol and an effective vaccine to prevent it [57,59]. Moreover, existing drugs approved by regulatory agencies such as EMA and FDA could be chosen for novel antiviral treatments, often eliminating the necessity for pharmacokinetic and toxicological investigations [60]. For drug repurposing, the clinical trials of the approved drugs for their new application require a fundamental understanding of pharmacokinetics and pharmacodynamics [61]. However, the large amount of Phase IV and post-marketing data offer an extensive understanding in terms of drug candidate selection for the new application [62].

The thiopurine AZA/6-MP are used widely and have a moderately narrow therapeutic index, and it is important to recognize the risk factors that may lead to toxicity, especially myelosuppression. One risk factor is the genetic polymorphism of the enzyme TPMT, which is involved in the metabolism of the thiopurine drugs; patients with intermediate or deficient TPMT activity are at risk for excessive toxicity after receiving standard doses of thiopurine medications. Recent insights into pharmacogenomics of thiopurine drugs have led to labeling revisions. Adverse drug reactions such as influenza-like symptoms, rash, and pancreatitis are not explained by TPMT deficiency. Polymorphisms in the ITPase gene may account for these non–TPMT-related adverse effects [63]. There, a strong role from AZA as a procarcinogenic drug has also been reported, both in organ transplant recipients and in other immune-mediated inflammatory diseases, particularly IBD [64]. Likewise, a mutational signature analysis on cutaneous squamous cell carcinoma (cSCC) primary tumors has revealed the presence of a novel signature, whose incidence correlates with chronic exposure to AZA increasing the risk for cutaneous malignancy in AZA-treated patients [65]. Although adverse effects are mainly reported in chronic use, the understanding of acute adverse effects is critical for the wide implementation and use of thiopurines as antivirals, in addition to the dose and pharmacokinetic understanding of the antiviral pathway for different viruses.

In addition, molecular docking studies combined with medicinal chemistry can be carried out that seek to improve the activity and pharmacokinetic properties of AZA and its derivatives. Therefore, it is crucial to advocate for the utilization of secure and efficient broad-spectrum repurposed drugs, such as AZA and its derivates, as antivirals. This approach aims to prevent the damages of viral diseases in both human and veterinary medicine, thereby broadening the spectrum of available medications to impede the spread and occurrence of emerging and re-emerging viral diseases.

## Figures and Tables

**Table 1:** List of viruses for which the antiviral activity of AZA and its derivatives has been demonstrated.

Virus	Family or Gender	Composite	Genome	Year	Ref
VZV	HERPESVIRIDAE	AZA	DNA	1990,1991	[41,42]
HSV	HERPESVIRIDAE	AZA	DNA	1990,1991	[41,42]
HCMV	HERPESVIRIDAE	AZA	DNA	1990,1991	[41,42]
BVDV	FLAVIVIRUS	AZA	Positive single-stranded RNA	2004	[43]
BVDV	FLAVIVIRUS	6-MP, 6-TI, 6-TGr, 6-MMPr	Positive single-stranded RNA	2008	[44]
SARS	CORONAVIRIDAE	6-MP, 6-TGN	Negative single-stranded RNA	2008	[45]
WNV	FLAVIVIRUS	6-MMPr	Positive single-stranded RNA	2011	[46]
DENV-2	FLAVIVIRUS	6-MMPr	Positive single-stranded RNA	2011	[46]
YFV	FLAVIVIRUS	6-MMPr	Positive single-stranded RNA	2011	[46]
MERS	CORONAVIRIDAE	6-MP, 6-TGN	Negative single-stranded RNA	2015	[47]
ZIKV	FLAVIVIRUS	6-MMPr	Positive single-stranded RNA	2017	[48]
CDV	MORBILLIVIRUS	6-MMPr	Negative single-stranded RNA	2017	[49]
RSV	PNEUMOVIRUS	AZA, 6-MP	Negative single-stranded RNA	2019	[50]
IAV	ORTHOMYXOVIRIDAE	6-TGN, 6-Tgo	Negative segmented RNA	2021	[51]
